# Aspectos Clínicos e de Sobrevida de Pacientes pós Implante de Valva Mecânica, com Ênfase em Trombose de Prótese Valvar

**DOI:** 10.36660/abc.20210544

**Published:** 2022-11-01

**Authors:** Fábio Tagliari, Marcelo Goulart Correia, Guilherme Dalcol Amorim, Alexandre Siciliano Colafranceschi, João Manoel Pedroso, Luiz Fernando Rodrigues, Thaisa Rodrigues Tagliari, Clara Weksler, Cristiane Lamas

**Affiliations:** 1 Instituto Nacional de Cardiologia Rio de Janeiro RJ Brasil Instituto Nacional de Cardiologia, Rio de Janeiro, RJ – Brasil; 2 Universidade Federal do Estado do Rio de Janeiro Rio de Janeiro RJ Brasil Universidade Federal do Estado do Rio de Janeiro, Rio de Janeiro, RJ – Brasil; 3 Universidade do Grande Rio Rio de Janeiro RJ Brasil Universidade do Grande Rio (UNIGRANRIO), Rio de Janeiro, RJ – Brasil; 4 Fiocruz Instituto Nacional de Infectologia Evandro Chagas Rio de Janeiro RJ Brasil Instituto Nacional de Infectologia Evandro Chagas, Fiocruz, Rio de Janeiro, RJ – Brasil

**Keywords:** Anticoagulantes, Análise de Sobrevida, Brasil, Implante de Prótese de Valva Cardíaca, Trombose

## Abstract

**Fundamento:**

As doenças oro-valvares têm prevalência mundial expressiva, e a cirurgia de troca valvar melhorou a sobrevida dos pacientes.

**Objetivos:**

Descrever aspectos clínico-laboratoriais dos pacientes submetidos a implante valvar mecânico e determinar a incidência de trombose de próteses valvares (TPV).

**Métodos:**

Estudo de coorte retrospectivo com seguimento até nove anos; as variáveis de estudo foram buscadas em prontuários físicos e eletrônicos. Os cálculos foram realizados pelo programa Jamovi 1.2.2.; p<0,05 foi considerado estatisticamente significante. Foram construídas curvas de Kaplan Meier, e realizada análise de regressão de Cox para fatores relacionados à mortalidade.

**Resultados:**

Foram incluídos 473 pacientes com média de idade de 46,9 ±11,3 anos. A doença reumática foi a principal etiologia. Em média de acompanhamento de 4,43 anos, a mortalidade foi de 16,1%. Pacientes com implantes de próteses na posição aórtica tiveram sobrevida melhor que os portadores em posição mitro-aórtica (p=0,026). Entre os fatores ajustados para mortalidade, apenas classe funcional e insuficiência renal crônica apresentaram significância estatística. A incidência de TPV foi de 0,24/100 pacientes/ano, com primeiro evento após 1000 dias da cirurgia. Tabagismo e *pannus* foram estatisticamente associados a TPV. Não houve diferenças na variabilidade de INR entre pacientes com e sem trombose por posição protética, mas houve diferença estatística no INR pré-evento trombótico comparado aos que não apresentaram trombose (INR= 2,20[1,80-2,20] vs 2,80[2,20-3,40]; p= 0,040). Identificamos 4,4% de acidentes vasculares cerebrais e 5,2% de sangramentos.

**Conclusões:**

A população mostrou-se jovem e valvopatia reumática foi frequente. A frequência de TPV foi semelhante à descrita na literatura, apesar da baixa renda e escolaridade da amostra.

## Introdução

As doenças orovalvares apresentam prevalência expressiva, afetando mais de 100 milhões de pessoas em todo o mundo.^1^ Em países em desenvolvimento, como o Brasil, as valvopatias representam uma significativa parcela das internações por doença cardiovascular. Nesses países, a doença valvar reumática incide de maneira preponderante^2^ e é um problema de saúde pública, com impacto socioeconômico, atingindo sobremaneira a população mais humilde e jovem.^3^

Embora o manejo clínico seja eficaz na maioria dos casos, em casos mais graves de doença valvar, a terapia cirúrgica para reparo ou troca da valva afetada tem indicação absoluta. Na substituição de válvula cardíaca, são usadas próteses constituídas por material biológico ou não-biológico (mecânicas), sendo as últimas de extensa durabilidade e largamente utilizadas em pacientes mais jovens.^4,5^ Contudo, comparativamente, as válvulas protéticas mecânicas associam-se à maior probabilidade de formação de trombos e eventos tromboembólicos devido a suas características físicas, com incidência total de trombose valvar mecânica de 0,4 por 100 pacientes por ano. Importante destacar que a trombose de prótese mecânica em posição mitral é de 0,5 por 100 pacientes por ano, aproximadamente cinco vezes mais frequente que em posição aórtica (0,1 por 100 pacientes por ano).^6^

Neste sentido, o acompanhamento clínico pós-operatório deve ser rigoroso visto que a anticoagulação inadequada pode favorecer à trombose de prótese com consequente disfunção, com ou sem tromboembolismo.^7^ Logo, a preferência pela válvula mecânica deve ser individualizada mesmo em pacientes mais jovens, considerando as variáveis: risco de sangramento, nível educacional e de compreensão do paciente, seu local de moradia e distância dos recursos médico-hospitalares na região, desejo do paciente quanto ao tipo de prótese e desejo de engravidar nas mulheres.^4^

No Brasil, são escassos os estudos descrevendo trombose protética e seu manejo.^8,9^ Portanto, o presente trabalho pretende descrever as características demográficas, clínicas, cirúrgicas e os desfechos de pacientes submetidos a implante de prótese valvar mecânica em uma instituição pública terciária referência em cardiologia de alta complexidade no Sistema Único de Saúde, com ênfase na incidência de trombose de prótese mecânica.

## Métodos

### Desenho do estudo

Este é um estudo do tipo coorte retrospectivo. Os pacientes foram identificados no banco de dados do Serviço de Doenças Orovalvares e no Registro de Cirurgias do Serviço de Cirurgia de um hospital terciário. As variáveis de estudo foram coletadas dos prontuários físicos e eletrônicos. Todos os pacientes operados que foram seguidos na instituição tiveram a verificação do INR (razão normalizada internacional) a cada quatro a seis semanas no ambulatório de anticoagulação. Pacientes que vinham em acompanhamento regular, mas faltaram por mais que 12 meses seguidos, foram pesquisados quanto à possibilidade de óbito junto ao Portal Extrajudicial do Estado do Rio de Janeiro, uma vez que todos os pacientes com prótese valvar mecânica têm consultas agendadas pelo menos semestralmente no ambulatório.

### População de estudo

Foram estudados todos os pacientes adultos que tiveram próteses valvares mecânicas implantadas no Instituto Nacional de Cardiologia, Rio de Janeiro, de janeiro de 2011 a dezembro de 2017.

### Variáveis de estudo

Variáveis buscadas foram sexo, idade, condição socioeconômica, comorbidades, medicações em uso, presença de fibrilação atrial; local de origem por regiões do Estado do Rio de Janeiro, etiologia e tipo de lesão valvar original, classe funcional (NYHA) na consulta ambulatorial mais recente, dados funcionais e hemodinâmicos de ecocardiografia pós implante valvar e o mais atual, marcas e posições valvares implantadas, nível de anticoagulação pela mensuração do tempo de ativação da protrombina (TAP) e valores de INR seriados nos últimos seis meses ou prévios ao diagnóstico de trombose valvar ou óbito. Foi calculada a incidência de trombose valvar, de acidente vascular cerebral e sangramentos, e avaliado tipo de intervenção e presença ou não de *pannus* associado à trombose.

### Definições operacionais

Trombose de prótese valvar (TPV) foi definida como qualquer trombo, na ausência de infecção, inserido ou perto da prótese valvar, ocluindo parte do fluxo sanguíneo ou interferindo com a função valvar.

Novo episódio TPV foi definido como aquele ocorrido mais de três meses após intervenção terapêutica em que foi documentada resolução do trombo, confirmada pela avaliação clínica e por métodos complementares.

Sangramento grave foi definido como aquele em que há risco iminente de morte, com intervenção cirúrgica de urgência ou não, com uso de hemoderivados ou não; sangramentos maiores foram definidos como aqueles potencialmente graves, com internação obrigatória, porém de conduta predominantemente conservadora, com uso ou não de hemoderivados.

### Análise de dados

Os dados foram expressos como frequências (variáveis categóricas), médias e desvio padrão (variáveis contínuas com distribuição normal) ou mediana e intervalo interquartil (variáveis contínuas sem distribuição normal). A análise estatística foi realizada utilizando-se o software específico Jamovi, versão 1.2.2. As variáveis categóricas foram analisadas pelos testes do qui-quadrado e exato de Fisher. O teste t de Student não pareado foi usado para comparação entre as variáveis contínuas com distribuição normal, e o teste de Mann-Whitney para as variáveis contínuas sem distribuição normal. Foi utilizado o teste de Shapiro-Wilk para verificar a normalidade da distribuição. A comparação entre as médias em mais de dois momentos foi realizada por análise da variância (ANOVA) para medidas repetidas. A análise de eventos (óbito e trombose de prótese) foi realizada por meio da Curva de Kaplan-Meier. O valor de p<0,05 foi considerado estatisticamente significante. Os efeitos de variáveis sobre a sobrevida após implante valvar foram avaliados usando-se índices de risco (*hazard ratio*, HR) ajustados (aHR) e seus correspondentes intervalos de confiança de 95% (IC), que foram estimados por meio dos modelos de regressão múltipla de risco proporcional de Cox. As suposições de risco proporcional para os ajustes dos modelos de regressão Cox foram testadas usando análises de correlação e testes de qui-quadrado baseados em resíduos Schoenfeld escalonados e tempos de sobrevivência transformados.

### Considerações éticas

O estudo foi aprovado pelo Comitê de Ética e Pesquisa do Instituto Nacional de Cardiologia em 01/08/18, sob o número CAAE: 87442918.3.0000.5272, parecer no. 2.793.851.

### Resultados

**Figura 1:**
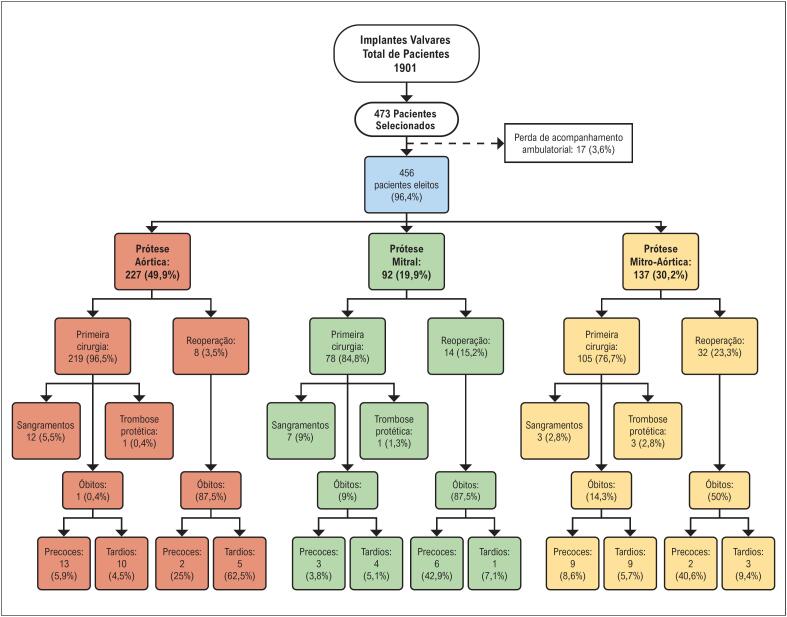
Fluxograma de inclusão de pacientes com prótese valvar e desfechos conforme localização da prótese valvar.

No período de estudo, o total de trocas valvares na instituição foi de 1901 implantes, entre próteses biológicas e mecânicas. Um total de 473 (24,9%) foram implantes de próteses metálicas (Figura 1) e, desses, 456 foram acompanhados até dezembro de 2019, com um seguimento médio por paciente de 4,4 anos. Dezessete pacientes não continuaram seguimento na instituição.

Foram implantadas 609 próteses mecânicas no total, sendo 49,9% na posição aórtica, 30,2% na posição mitral e aórtica, e 19,9% na mitral, das marcas St. Jude^R^ (Minneapolis, USA), (n=465, 74,2%), ATS Medical^R^ (Minnesota, USA) (n=159, 25,4%), Carbomedics^R^ (Austin, USA) (n=1, 0,2%); modelos não foram especificados em 0,3% dos casos (n=2).

A Tabela 1 apresenta as características clínico-demográficas dos 473 pacientes do estudo de acordo com a posição protética. A média de idade foi de 46,9 ^±^ 11,3 anos. A maior parte dos pacientes possuía o ensino fundamental (completo ou incompleto), e renda de até três salários mínimos. Dentre as comorbidades, a hipertensão arterial sistêmica essencial foi a mais frequente, em mais da metade dos casos, seguida de dislipidemia em cerca de um quarto, e diabetes mellitus tipo 2. De todos os pacientes, 46,5% eram do município do Rio de Janeiro, e 37,3% eram oriundos de municípios da Baixada Fluminense.

**Tabela 1:** Características demográficas e clínicas dos pacientes submetidos à troca valvar mecânica, Janeiro de 2011 a Dezembro de 2017

Variáveis			Aórtica (n=236)	Mitral (n=96)	Mitro-aórtica (n=141)	p valor
(n = 473)	Masculino		148(62,7%)	31 (32,3%)	51 (36,2%)	p<0,01
	Feminino		88 (37,3%)	65 (67,7%)	90 (63,8%)	
**Idade em anos** (n = 473)	< 20		0	0	0	p=0,015
	20-29		6 (2,5%)	2 (2,1%)	3 (2,1%)	
	30-39		26 (11,0%)	7 (7,3%)	14 (9,9%)	
	40-49		35 (14,8%)	19 (19,8%)	43 (30,5%)	
	50-59		70 (29,6%)	36 (37,5%)	46 (32,6%)	
	60-69		82 (34,7%)	25 (26,0%)	32 (22,7%)	
	≥ 70		17 (7,2%)	6 (6,2%)	3 (2,1%)	
**Escolaridade** (n = 382)	Analfabeto		1 (0,42%)	0	1 (0,7%)	p=0,003
	Ensino Fundamental		95 (40,2%)	55 (57,3%)	77 (54,6%)	
	Ensino Médio		73 (30,9%)	16 (16,6%)	32 (22,7%)	
	Ensino Superior		22 (9,3%)	1 (1,0%)	9 (6,3%)	
**Renda mensal** (em salários mínimos) (n = 153)	Até 1		23	1	15	p=0,697
	De 1 a 2		19	2	19	
	De 2 a 3		17	0	12	
	> 3		29	1	15	
**Comorbidades associadas**	HAS	**Sim**	143(60,6%)	48 (50,5%)	66 (46,8%)	p=0,017
		**Não**	93 (39,4%)	47 (49,5%)	75 (53,2%)	
	DLP	**Sim**	78 (33,6%)	25 (26,6%)	19 (13,7%)	p<0,001
		**Não**	154(66,4%)	69 (73,4%)	120 (86,3%)	
	DM2	**Sim**	31 (13,6%)	16 (17,2%)	8 (5,8%)	p=0,020
		**Não**	197(86,4%)	77 (82,8%)	129 (94,2%)	
	Tabagismo	**Sim**	19 (8,0%)	3(3,1%)	10 (7,1%)	p=0,273
		**Não**	217(92%)	92 (96,9%)	131 (92,9%)	
	AVE prévio	**Sim**	5 (2,1%)	9(9,5%)	12 (8,5%)	p=0,006
		**Não**	229(97,9%)	86 (90,5%)	129 (91,5%)	
	AIT	**Sim**	2 (0,8%)	0	1 (0,7%)	p=0,672
		**Não**	232(99,2%)	95 (100%)	139 (99,3%)	
	DRC	**Sim**	4 (1,7%)	7 (7,3%)	5 (3,6%)	p=0,038
		**Não**	232(98,3%)	89 (92,7%)	135 (96,4%)	
	DPOC	**Sim**	12 (5,1%)	2 (2,1%)	3 (2,1%)	p=0,224
		**Não**	223(94,9%)	93 (97,9%)	137 (97,9%)	
	Etilismo	**Sim**	1 (0,4%)	0	0	p=0,605
		**Não**	233(99,6%)	95 (100%)	140 (100%)	
	Hepatopatias	**Sim**	0	0	3 (2,1%)	p=0,029
		**Não**	233 (100%)	94 (100%)	137(97,9%)	
**Fibrilação Atrial** (n = 473)	Presente		19 (8,0%)	52 (54,2%)	62 (44%)	p<0,001
	Ausente		217 (92%)	44 (45,8%)	79 (56%)	

HAS: hipertensão arterial sistêmica; DLP: dislipidemia; DM2: diabetes mellitus tipo 2; AVE: acidente vascular encefálico; AIT: ataque isquêmico transitório; DRC: doença renal crônica; DPOC: doença pulmonar obstrutiva crônica.

Nota: Os números nas variáveis escolaridade, renda mensal, e comorbidades correspondem àqueles em que a informação estava disponível nos prontuários, visto ser este um estudo retrospectivo

**Figura 2:**
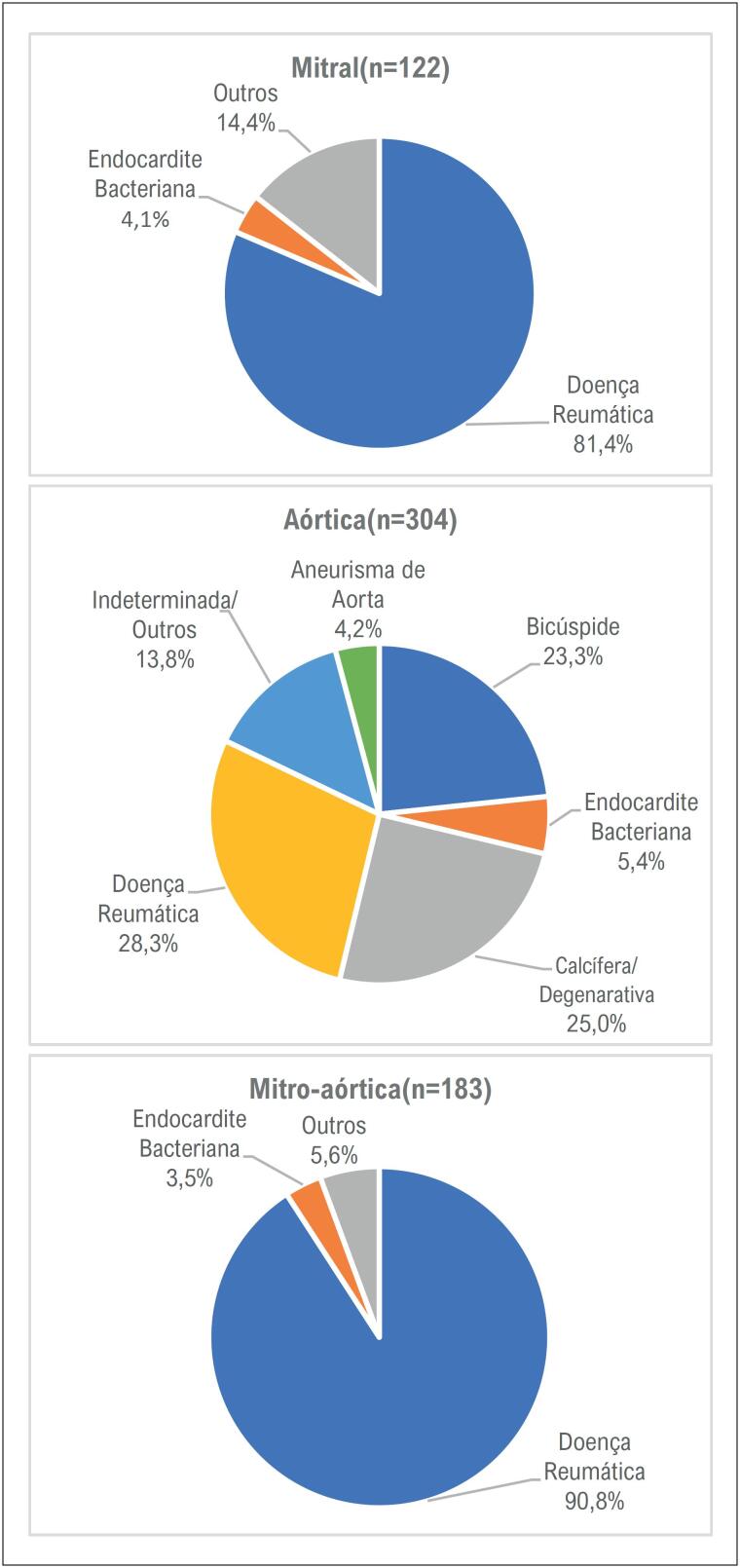
Etiologia de doença valvar de base de acordo com a posição da prótese mecânica inserida.

A doença cardíaca reumática foi a etiologia predominante, em 57,7% dos casos, seguida da doença valvar degenerativa (12,9%) e válvula aórtica bicúspide (12,1%). A endocardite infecciosa apresentou-se como a principal etiologia secundária, motivando a segunda troca valvar em 24 (5,1%) dos casos. A Figura 2 mostra as etiologias por categorias mitral, aórtica e mitro-aórtica.

Foram encontradas como lesões graves 107 casos de estenose aórtica (45,9%), 93 (39,9%) de insuficiência aórtica e 16 (6,8%) de dupla lesão aórtica; estenose mitral grave em 41 (44%), insuficiência mitral em 30 (32,2%) e dupla lesão mitral em 8 (8,6%).

A classe funcional (CF) da *New York Heart Association* (NYHA) de 422 pacientes relatada na última visita ambulatorial foi CF I em 323 (76,5%); CF II em 85 (20,1%), CF III em 12 (2,8%), e CF IV em apenas dois (0,5%).

A Tabela 2 mostra os dados ecocardiográficos de exames realizados após o implante valvar, e dados mais recentes obtidos no acompanhamento do paciente. Na comparação do ecocardiograma realizado logo após o implante cirúrgico com o exame mais recente, as próteses em posição aórtica apresentaram melhora de todos os parâmetros hemodinâmicos (p<0,001). Nas posições mitral e mitro-aórtica, houve melhora na fração de ejeção média e gradiente pressórico médio entre átrio esquerdo e ventrículo esquerdo.

**Tabela 2:** Comparação entre parâmetros ecocardiográficos pós-implante valvar e o mais atual conforme posição da prótese mecânica

		PRÉ ALTA HOSPITALAR [Média (DP) ou mediana (IIQ)]	EXAME MAIS RECENTE (Média (DP) ou mediana [IIQ])	Valor de p
**Prótese posição Aórtica** (n= 233)				
	FEVE (%)	54,1 (14,7)	62,6 (12,0)	< 0,001
	Gradiente VE/AO máximo (mmHg)	32,0 [25,0 - 41,8]	26,0 [20,0 - 34,0]	< 0,001
	Gradiente VE/AO médio (mmHg)	18,0 [13,0 - 23,0]	14,0 [11,5 - 15,8]	< 0,001
**Prótese posição Mitral** (n= 93)				
	FEVE (%)	54,2 (2,7)	56,8 (13,4)	0,028
	Gradiente AE/VE máximo (mmHg)	13,3 (4,55)	26,6 (4,04)	0,837
	Gradiente AE/VE médio (mmHg)	5,0 [4,0 - 6,0]	4,0 [4,0 - 5,0]	0,036
**Prótese nas posições Mitro-aórtico** (n= 141)				
	FEVE (%)	55,5 (14,2)	61,2 (12,7)	<0,001
	Gradiente VE/AO máximo (mmHg)	30,0 [23,0 - 39,5]	29,0 [21,0 -40,5]	0,477
	Gradiente VE/AO médio (mmHg)	17,0 [11,0 -23,0]	16,0 [11,0 -22,3]	0,642
	Gradiente AE/VE máximo (mmHg)	12,4 (5,05)	12,4 (5,1)	0,749
	Gradiente AE/VE médio (mmHg)	5,0 [3,0 - 6,0]	4,0 [3,0 - 5,0]	0,003

IIQ: intervalo interquartil; FE: fração de ejeção do ventrículo esquerdo; AE: átrio esquerdo; VE: ventrículo esquerdo; AO: aorta; Gradiente: gradiente pressórico; teste t de Student não pareado e o teste de Mann-Whitney.

**Figura 3:**
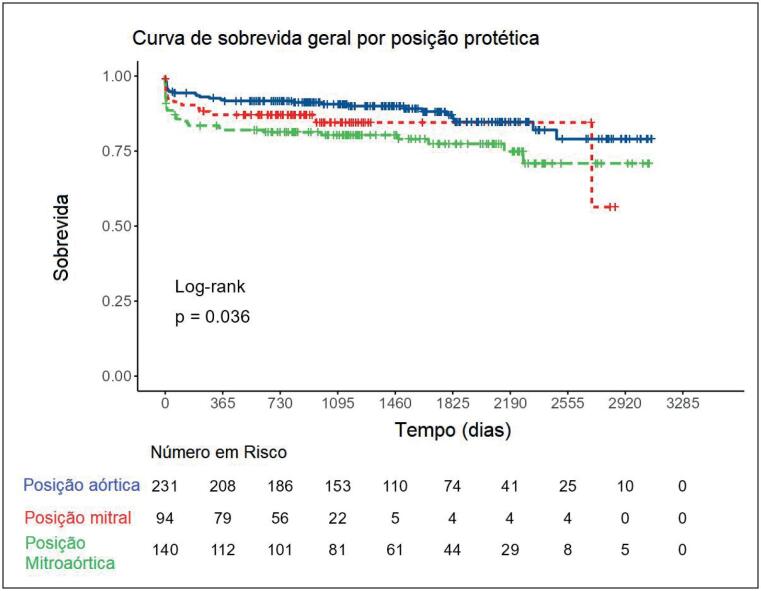
Curva de sobrevida por posição valvar em pacientes submetidos a troca valvar, Instituto Nacional de Cardiologia, Rio de Janeiro, janeiro de 2011 a dezembro de 2017.

A Figura 3 mostra a curva de sobrevida das próteses implantadas estratificadas por posição. Pacientes com implantes de próteses na posição aórtica tiveram sobrevida maior que os portadores em posição mitro-aórtica (p=0,026). Não houve diferenças entre as demais comparações. Na curva de sobrevida, não houve diferenças estatísticas entre as faixas etárias e gênero (Figuras Suplementares 1 e 2). Quando analisamos separadamente a sobrevida nos pacientes com doença valvar de base reumática, não houve diferença entre as posições valvares. No entanto, em relação aos não reumáticos, houve diferença de sobrevida entre as posições, mas o número de indivíduos que operaram a válvula mitral ou a mitral e aórtica foi muito pequeno, sendo maior o número de indivíduos operados na posição aórtica (n=127) (Figuras Suplementares 3 e 4). Quando comparados reumáticos e não reumáticos para a posição aórtica, não houve diferença na sobrevida (Figura Suplementar 5).

A regressão de Cox para variáveis relacionadas à sobrevida mostrou que o principal fator relacionado a óbito foi classe funcional após o implante valvar. Para CF II, o aHR foi de 5,18 (2,17-12,39; p<0,001), para CF III, aHR foi 41,13 (14,95-113,15, p<0,001) e para CF IV, aHR=200,48 (21,60-1861,12, p<0,001). Outro fator associado foi a presença de insuficiência renal crônica, cujo aHR foi de 3,52 (1,12-11,09; p=0,032). As posições de troca valvar mecânica não tiveram diferença estatisticamente significante após o ajuste. Esses resultados são apresentados na Figura Suplementar 6.

Houve 76 (16,1%) óbitos por todas as causas; 36 (7,4%) pacientes morreram em 30 dias. Considerando-se óbitos por diferentes posições das próteses, a mortalidade em pacientes com próteses aórticas foi de 6%, em posição mitral de 8,2% e em posição mitro-aórtica de 14,2%. As causas de morte mais frequentemente encontradas foram choque cardiogênico (R57.0), choque hipovolêmico e coagulação intravascular disseminada (R57.1 e D65). Dos 17 pacientes que foram perdidos para acompanhamento na instituição, um foi a óbito segundo Portal Extrajudicial do Estado do Rio de Janeiro, contudo não estava explicitada a *causa mortis*.

A incidência total de trombose por paciente foi de 1,1% (0,24 por 100 pacientes/ano), com sete eventos trombóticos em cinco pacientes. A ocorrência de trombose de prótese mecânica ocorreu em tempo tardio ao implante valvar protético, sendo o primeiro evento a partir do quinto ano após cirurgia. O Quadro 1 apresenta detalhadamente os dados dos pacientes com TPV.

**Quadro 1:** Aspectos clínicos e ecocardiográficos, e desfechos dos cinco pacientes, em sete eventos, diagnosticados com trombose de prótese valvar mecânica

Paciente	1	2	3	4	5
Idade	43	44	52	38	54
Sexo	Masculino	Feminino	Masculino	Feminino	Feminino
Escolaridade	Fundamental	Fundamental	Fundamental	Superior	Fundamental
Renda Familiar	2 sal.	1 sal.	5 sal.	5 sal.	4 sal.
Rio de Janeiro	Sim	Sim	Sim	Não	Sim
Etiologia primária	Reumática	Reumática	Reumática	Reumática	Congênita/Bicúspide
Próteses	Mitral 27	Aórtica 18 Mitral 27	Aórtica 21 Mitral 27	Aórtica 21 Mitral 29	Aórtica 19
Marca da prótese	ATS	ATS	St Jude ATS	St Jude St Jude	St Jude
Fibrilação atrial	Sim	Não	Sim	Não	Não
Tempo implante x evento(s) trombose (s)	8 anos	A – 5 anos B – 7 anos	A – 5 anos B – 2 anos	6 anos	5 anos
Tabagismo	Não	Não	Sim	Sim	Não
CF (NYHA)	III	A – I B – III	A – II B – IV	III	II
Acompanhamento	Atual	Atual	Atual	Atual	Não atual (óbito)
Disfunção de VE	Sim	Sim	Não	Não	Não
Pannus associado	Não	Sim	Sim	Sim	Sim
Tratamento	HNF+TT	A – HNF+TT B – Cirurgia	A – HNF+TT B – Cirurgia	Cirurgia	Cirurgia

A e B referem-se ao primeiro e ao segundo episódio de trombose valvar protética, respectivamente, em um mesmo paciente; CF: classe funcional (NYHA); ATS: prótese valvar mecânica bifolheto da empresa Medtronic; St. Jude: prótese valvar mecânica bifolheto da empresa Abbott; HNF: heparina não fracionada; TT: terapia trombolítica; Disfunção de VE (ventrículo esquerdo): Grau de insuficiência do ventrículo esquerdo caracterizada como fração de ejeção (FE) calculada pelo método de Teichholz igual ou menor que 52% de acordo com a American Society of Echocardiography; Sal: salário(s).

Na Tabela 3, é apresentada a comparação da variabilidade mensal do valor de INR, do mês mais recente (INR6) ao mais distante (INR1), entre os pacientes com TPV e aqueles sem TPV. Não houve variabilidade significante do INR entre esses grupos ao longo do tempo. A ausência de valores entre os parênteses exprime não haver desvio padrão pela presença de apenas um paciente com trombose na amostra. De modo semelhante, não houve diferença na variabilidade do INR quando os pacientes com sangramento foram comparados aos que não tiveram sangramento.

**Tabela 3:** Variabilidade do valor de INR entre pacientes com e sem trombose de prótese valvar

Trombose	INR mês 1 (n=52)	INR mês 2 (n=144)	INR mês 3 (n=256)	INR mês 4 (n=335)	INR mês 5 (n=381)	INR mês 6 (n=407)	Valor de p
**Sim** (n=5)	4,70 (-)	1,50 (-)	2,37 (0,61)	3,00 (2,02)	2,30 (0,673)	2,06 (0,42)	0,392
**Não** (n=451)	3,09 (1,45)	3,15 (1,30)	2,97 (1,12)	2,97 (1,23)	2,82 (1,00)	2,94 (1,26)	

Valores estão expressos em média (±DP); INR: *international normalized ratio*; teste ANOVA para medidas repetidas.

A Tabela 4 mostra os valores de INR nas seis coletas anteriores ao evento de trombose, nos pacientes que evoluíram com TPV, e nas seis coletas anteriores à última consulta, nos que não tiveram TPV. Não houve diferença estatística entre os valores de INR entre os pacientes com trombose e sem trombose em relação a posição do implante protético valvar. A ausência de valores em determinados meses dos INRs das próteses em posição mitral e aórtica impossibilita cálculos para efeito comparativo. O INR dos pacientes que evoluíram com trombose foi significativamente menor que de pacientes que não apresentaram trombose [mediana 2,20 (1,80-2,20) e 2,80 (2,20-3,40), respectivamente, p=0,04] no mês anterior ao evento (INR mês 6), independentemente da posição protética. Quando separamos por posição protética, não houve diferença estatisticamente significativa entre os INR dos pacientes que tiveram e não tiveram TVP (Tabela 4). Foi observada associação entre TPV e tabagismo (2/5 dentre os pacientes com TVP vs. 27/441 dentre os sem TPV). A presença de *pannus* foi diagnosticada no ato cirúrgico em 11 pacientes, sendo quatro associados à trombose (p<0,001). Dos sete eventos de TPV detectados em cinco pacientes, ocorreu um óbito em pós-operatório imediato de cirurgia de troca valvar (Quadro 1).

**Tabela 4:** Análise comparativa dos valores dos INR mensais entre os pacientes com trombose e sem trombose conforme posição da prótese mecânica implantada

Prótese/Posição		Mitral (alvo INR=2,5 a 3,5)*		Mitro-aórtica (alvo INR=3)*		Aórtica (Alvo 2,5 a 3,5)*	
Trombose		Sim	Não	Sim	Não	Sim	Não
**INR mês 1**		---------	---------	4,70 [4,70 - 4,70]	2,80 [2,55 - 4,00]	---------	---------
	Valor de p	---------	---------	0,193		---------	---------
**INR mês 2**		---------	---------	1,50 [1,50 - 1,50]	3,15 [2,32 -3,60]	---------	---------
	Valor de p	---------	---------	0,163		---------	---------
**INR mês 3**		---------	---------	2,70 [2,60 - 2,80]	3,00 [2,30 - 3,50]	1,70 [1,70-1,70]	2,70 [2,10-3,30]
	Valor de p	---------	---------	0,600		0,161	
**INR mês 4**		---------	---------	1,85 [1,68 - 2,03]	2,95 [2,40 - 3,73]	5,30 [5,30-5,30]	2,60 [2,20-3,20]
	Valor de p	---------	---------	0,063		0,098	
**INR mês 5**		1,30 (-)	2,94 (0,981)	2,60 (0,141)	3,05 (1,10)	2,70 [2,70-2,70]	2,60 [2,00-3,10]
	Valor de p	0,100		0,565		0,865	
**INR mês 6**		2,60 [2,60-2,60]	3,00 [2,2-3,70]	2,20 [1,85 - 2,2]	2,90 [2,20 - 3,50]	1,80 [1,80-1,80]	2,60 [2,10-3,30]
	Valor de p	0,71		0,073		0,211	

Valores de Referência para INR de acordo com Nishimura et al.^27^; teste t de Student não pareado e o teste de Mann-Whitney; INR: *international normalized ratio*.

Com relação a eventos hemorrágicos, foram identificados 23 pacientes com sangramento, sendo oito (1,7%) classificados como grave, e 15 (3,5%) como maior. A taxa de sangramento foi de 1,02 por 100 pacientes por ano. Ocorreram dois óbitos em pacientes com sangramento grave, um por tamponamento cardíaco e outro por acidente vascular encefálico hemorrágico. Acidentes vasculares cerebrais isquêmicos ocorreram em 4,4% da amostra, com incidência de 0,86 por 100 pacientes por ano.

Foram identificados cinco pacientes com *leak* paraprotético. Dois deles haviam tido endocardite infecciosa como motivo da troca valvar mecânica, dois outros fizeram *leak* após retroca valvar, e um deles era um reumático com doença mitro-aórtica. Dos cinco, apenas um precisou ser operado por conta do *leak* e foi a óbito; em dois foi feita oclusão percutânea do *leak.*

## Discussão

O presente estudo avaliou as características demográficas, clínicas, cirúrgicas e os desfechos de pacientes submetidos a implante de prótese valvar mecânica em uma instituição pública terciária de referência em cardiologia de alta complexidade no Sistema Único de Saúde, com ênfase na incidência de trombose de prótese mecânica. Como resultados principais, observou-se nessa amostra de 473 pacientes proporção semelhante entre os sexos, em consonância com a literatura mais recente,^10,11^ mas diferente do estudo de Brandão et al.,^9^ publicado há três décadas, no estado de São Paulo, em que o gênero masculino correspondeu a 64,3%. O perfil socioeconômico revelou-se baixo, com a maioria apresentando renda mensal de até três salários mínimos e ensino fundamental completo ou incompleto. A média de idade de nossos pacientes foi 47 anos, mais baixa que os descritos na literatura internacional,^12–14^ mas semelhante às médias de artigos brasileiros,^10,11^ o que se dá essencialmente por ser a etiologia reumática a mais frequente causa de troca valvar no SUS. A maior parte dos pacientes era oriunda do município do Rio de Janeiro, seguida da região metropolitana I (Baixada Fluminense). A importância do local de moradia diz respeito à possível facilidade de acesso para um bom seguimento ambulatorial da anticoagulação.

A doença valvar reumática foi a mais frequente etiologia primária, acometendo mais da metade dos pacientes. A predominância da valvopatia reumática foi semelhante a dados nacionais, como em estudo no município de Salvador e em artigo sobre dados de cirurgia de troca valvar nacional do SUS, contrastando com países desenvolvidos.^1,2,7^ Dados da literatura brasileira, de populações com perfis semelhantes em vários aspectos à da nossa amostra, mostram uma prevalência de cirurgia em posição mitral, diferentemente deste estudo, que foi em posição aórtica. Acreditamos que múltiplos fatores possam ser responsáveis por essa diferença, sendo os mais importantes i) a valvopatia reumática frequentemente resulta em estenose mitral sobretudo em indivíduos do sexo feminino; neste grupo, há a preferência de implante de biopróteses na fase reprodutiva das mulheres; ii) para a valvopatia mitral, há a possibilidade de intervenção por valvuloplastia mitral percutânea com balão ou de comissurotomia cirúrgica (sem troca); na válvula aórtica reumática, isso não é possível. Em nossa amostra, de fato, houve casos de implante mitral mecânico prévio à data do estudo e, na época do estudo, o implante realizado se deu em posição aórtica apenas. Dentre os 67 pacientes reumáticos que tiveram apenas a válvula aórtica trocada, a comissurotomia mitral cirúrgica associada ao implante valvar aórtico mecânico foi realizada em sete (10,4%) pacientes. Havia lesões reumáticas mitrais leves associadas ao implante aórtico em 34 (50,7%) pacientes, e em dois pacientes, observaram-se lesões mitrais moderadas concomitantes, que não sofreram intervenção no ato cirúrgico. As marcas ATS e St. Jude foram quase que exclusivas nos implantes cirúrgicos. Verificamos melhor sobrevida nos pacientes com implante na posição aórtica em comparação àqueles com dupla troca mitro-aórtica (p=0,026), o que corrobora outros estudos.^13,15^ Não houve diferenças de sobrevida quanto ao gênero e idade embora somente 10 pacientes (17,1%) tinham mais que 65 anos em nossa amostra. Nossos resultados foram diferentes de um estudo brasileiro^10^ que apresentou taxa de sobrevida superior do sexo feminino em relação ao sexo masculino no primeiro e quinto ano de seguimento.^10^ Pela análise multivariada, os fatores fortemente relacionados a óbito pós troca valvar foram status funcional cardíaco no seguimento ambulatorial e a presença de insuficiência renal crônica como comorbidade.

Nosso estudo mostrou mortalidade geral de 16%, sendo a de 30 dias de 7,4%, sendo a mais elevada na posição mitro-aórtica. No estudo japonês de Tominaga et al.,^16^ publicado em 2005, em um acompanhamento por 10 anos em pacientes portadores da prótese mecânica tipo bifolheto Carbomedics, os autores relataram mortalidade precoce (hospitalar) de 2,8%, sendo 1,2% para posição aórtica, 3,6% mitral e 3,8% mitro-aórtica. Um artigo suíço da década de 90^13^ e um estudo belga^14^ descrevem óbito intra-hospitalar de 5,7% e 5,2% respectivamente, taxas de mortalidade um pouco inferiores à nossa. Vale comentar que as populações de estudo são demograficamente muito diferentes, predominando idosos. Em estudo nacional^11^ de vários hospitais do SUS, verificou-se taxa de mortalidade de 22,1%, superior ao nosso; em um intervalo de dois anos, ocorreram 12,3% óbitos, desses 8,5% na posição aórtica, 12,2% mitral e 18,4% na posição mitro- aórtica. A sobrevida em seguimento em cinco anos em nosso trabalho foi de 83,4% versus 74,5% dos pacientes deste estudo brasileiro.^11^

Considerando a ocorrência de TPV, nossos dados vão ao encontro da literatura internacional, em que a taxa anual foi de 0,1 a 5,7% e 0,3-1,3%, respectivamente.^7,17^ Expresso de outra maneira, a incidência de trombose da nossa população foi discretamente inferior aos 0,31 por 100 pacientes por ano no estudo de Van Nooten et al., o que demonstra a baixa taxa deste evento em nosso centro. O tempo médio para o primeiro evento de trombose em nosso estudo foi superior ao encontrado em estudo canadense, em que foi de 39 meses.^18^ Considerando o nível socioeconômico de nossa população, a baixa taxa de trombose é um resultado positivo, o que nos encoraja a considerar a recomendação de próteses mecânicas para pacientes mais jovens com menos temor.

O tabagismo, que se mostrou associado à TPV de maneira estatisticamente significativa em nosso estudo, foi identificado por meio de prontuário médico, e sabidamente considerado um fator de hipercoagulabilidade secundária, contribuinte no mecanismo pró-trombótico, como descrito na literatura.^7^

O diagnóstico de TPV mecânica foi realizado por presunção clínica e utilização de métodos complementares disponíveis na instituição, sendo, para nós, o exame ecocardiográfico transesofágico o de maior importância. A radioscopia revelou-se de valia no auxílio diagnóstico complementar ao ecocardiograma. As estratégias de anticoagulação para próteses valvares mecânicas não estão bem definidas, havendo diferenças entre as diretrizes europeia e americana, por exemplo. A diretriz americana relata complicações relacionadas às flutuações nos valores de INR com o uso da varfarina, sugerindo a aplicação de um índice alvo único de INR.^19^ Para próteses em posição aórtica, INR alvo de 2,5; posição mitral ou posição aórtica com fatores de risco associados (fibrilação atrial, tromboembolismo prévio, disfunção ventricular esquerda, condição de hipercoagulabilidade), INR alvo de 3,0, associado ao uso de aspirina na dose de 75-100 mg (classe IA). Já a diretriz europeia determina o valor de INR de acordo com a trombogenicidade da prótese e fatores de risco associados do paciente, acrescentando a aspirina se houver doença aterosclerótica concomitante e/ou tromboembolismo apesar de INR adequado.^20^ Considerando valores de INR alvo nas diretrizes (entre 2,0 e 3,5), tanto do ponto de vista de efeito protetor antitrombótico como também o fator sangramento, nossos pacientes com próteses mecânicas em posição mitral ou aórtica se apresentaram no alvo referido em apenas 40,6% das vezes, com base nos resultados laboratoriais dos seis meses anteriores a sua última consulta. Há vários artigos que dissertam sobre o desafio que é manter o alvo de anticoagulação.^21–23^ A variação dos valores de INR nos seis últimos meses não mostrou diferenças estatisticamente significantes entre pacientes que não tiveram TPV e pacientes com TPV, tampouco quando comparados entre as posições protéticas. Já quando todos os pacientes com TPV foram comparados aos sem TPV, detectamos diferença estatística quanto ao último INR aferido. Embora esperássemos mais diferenças nessas comparações, isso não ocorreu possivelmente pelo pequeno número de eventos da amostra.

A formação de *pannus* associou-se de maneira estatisticamente significante à presença de trombos. Tal fato está em conformidade com a literatura, em que vários estudos sugeriram que a trombose ocorre com outras causas de disfunção de prótese valvar, como é o caso do crescimento do *pannus. A p*resença de *pannus* é um fator pró-trombótico.^7^

Com relação ao tratamento e desfechos dos pacientes com TPV, dos cinco pacientes, três pacientes utilizaram tratamento medicamentoso com heparina não fracionada seguindo-se a terapia trombolítica. Há publicações que dissertam sobre a efetividade da heparina não fracionada de longa duração, associada à anticoagulação oral, em prevenir eventos tromboembólicos precoces em trombos obstrutivos e pequenos (< 5 mm) após troca valvar mitral.^24^ Há consenso para seu uso somente em trombos não obstrutivos e pequenos no lado esquerdo do coração, ainda assim com efetividade reduzida, com recorrência de trombose em 16%.^25^ Já em trombos obstrutivos, comenta-se a inefetividade da heparina.^26^ Em nosso estudo, três pacientes submeteram-se à a terapia trombolítica com o ativador do plasminogênio tissular recombinante (rT-PA). Houve boa resposta em todos eles, com recorrência de trombose em dois pacientes em intervalo de tempo de sete meses e dois anos respectivamente. A nossa amostra diminuta não permite uma comparação precisa com dados da literatura. A heparina não fracionada se mostrou inefetiva em nossos pacientes. As diretrizes americanas e europeias definem que o tratamento cirúrgico é o de escolha nas TPV mecânica para pacientes com classe funcional NYHA III e IV, a menos que sejam de alto risco cirúrgico (classe IIa). Relatos de literatura têm reforçado a ideia de que a terapia trombolítica vem se consolidando cada vez mais no tratamento da TPV, entre eles os estudos TROIA e o PROMETEE,^27^ utilizando baixas doses em infusão lenta e ultralenta de rT-PA respectivamente. O ato cirúrgico na trombose de prótese mecânica é um procedimento de retroca valvar e seus riscos não podem ser subestimados; há relatos de índices de mortalidade em média de 12% nessas circunstâncias.^28^ Cirurgia na urgência ou emergência tem sido a estratégia de escolha, mas acompanha-se de significante mortalidade, variando de 7,1% a 69%, dependendo do estado funcional do paciente.^29^ Dois de nossos pacientes submeteram-se diretamente ao procedimento cirúrgico, sendo em um deles evidenciada gravidez e endocardite infecciosa associada ao quadro.

A taxa de sangramento em nosso estudo foi semelhante ao estudo italiano de 2018.^20^ Todos necessitaram internação hospitalar, e tratamento e acompanhamento específicos. Há estudo brasileiro de acompanhamento em 40,6 meses em pacientes que implantaram prótese valvar mecânica, observando incidência de sangramento de 0,95% por paciente-ano.^9^ Outro trabalho comenta que em paciente sob uso de varfarina com INR na faixa de 2,5-4,5, a possibilidade de sangramento é de 3% por paciente-ano.^29^

Em relação a eventos cerebrais vasculares isquêmicos, os valores são semelhantes aos de dados de literatura que relata 0,9 – 3,6 por 100 pacientes por ano.^21^

As limitações do estudo são ser um estudo de único centro, abrangendo população atendida pelo SUS, de modo que as conclusões podem não ser aplicáveis a outros centros. Por ser de caráter retrospectivo, alguns dados não foram passíveis de obtenção. Além disso, o número de eventos observado foi pequeno, como a própria trombose de prótese e sangramentos, sendo fator limitante para a análise de variáveis possivelmente associadas a esses eventos. Uma possível limitação seria a perda de eventos (TPV ou sangramento) que tenham ocorrido em outros hospitais.

## Conclusões

Nossa população de estudo é jovem e tem um histórico de trocas valvares cirúrgicas anteriores, tendo a etiologia reumática como a mais prevalente.

A incidência de TPV foi de 1,1% em conformidade com a literatura mundial, com eventos tardios após o implante. Dada a baixa escolaridade e renda de nossos pacientes, este achado foi positivo, o que nos encoraja a recomendar o implante de próteses mecânicas para pacientes neste perfil.

Fatores associados à TPV em todas as posições valvares avaliadas em conjunto foram INR fora do alvo, tabagismo e presença de *pannus.* Os fatores fortemente relacionados a óbito pós-troca valvar mecânica foram *status* funcional cardíaco no seguimento ambulatorial e presença de insuficiência renal crônica como comorbidade.

## *Material suplementar

Para informação adicional do Material Suplementar 1, por favor, clique aqui.

Para informação adicional do Material Suplementar 2, por favor, clique aqui.

Para informação adicional do Material Suplementar 3, por favor, clique aqui.

Para informação adicional do Material Suplementar 4, por favor, clique aqui.

Para informação adicional do Material Suplementar 5, por favor, clique aqui.

Para informação adicional do Material Suplementar 6, por favor, clique aqui.
