# Supplementary material for: Aspectos Clínicos e de Sobrevida de Pacientes pós Implante de Valva Mecânica, com Ênfase em Trombose de Prótese Valvar
Source: Arq Bras Cardiol. 2022 Nov 1;119(5):734–44. [Article in Portuguese] doi: 10.36660/abc.20210544 (PMC9750220; doi:10.36660/abc.20210544)
Supplement: Translation

# Translation: Clinical Features and Survival Analysis of Patients after Mechanical Heart Valve Replacement, with an Emphasis on Prosthetic Valve Thrombosis

**Authors:** Fábio Tagliari, Marcelo Goulart Correia, Guilherme Dalcol Amorim, Alexandre Siciliano Colafranceschi, João Manoel Pedroso, Luiz Fernando Rodrigues, Thaisa Rodrigues Tagliari, Clara Weksler, Cristiane Lamas

**Affiliations:** 1 Rio de Janeiro RJ Brazil Instituto Nacional de Cardiologia, Rio de Janeiro, RJ – Brazil; 2 Rio de Janeiro RJ Brazil Universidade Federal do Estado do Rio de Janeiro, Rio de Janeiro, RJ – Brazil; 3 Rio de Janeiro RJ Brazil Universidade do Grande Rio (UNIGRANRIO), Rio de Janeiro, RJ – Brazil; 4 Rio de Janeiro RJ Brazil Instituto Nacional de Infectologia Evandro Chagas, Fiocruz, Rio de Janeiro, RJ – Brazil

**Keywords:** Anticoagulants, Survival Analysis, Brazil, Heart Valve Prosthesis Implantation, Thrombosis

## Abstract

**Background:**

Valvular heart diseases are highly prevalent in the world, and surgical valve replacement has improved patients’ survival.

**Objectives:**

To describe clinical and laboratory data of patients undergoing mechanical valve replacement, and to determine the incidence of prosthetic valve thrombosis (PVT).

**Methods:**

Retrospective cohort study with a follow-up of up to nine years. The study variables were collected from conventional and electronic medical charts. Statistical calculations were performed using the Jamovi software version 1.2.2.; a p<0.05 was considered statistically significant. Kaplan Meier curves were constructed, and Cox regression analysis was performed for analysis of factors related to mortality.

**Results:**

A total of 473 patients were included, mean age of 46.9 ±11.3 years. Rheumatic disease was the most common etiology. In a mean follow-up period of 4.43 years, mortality rate was 16.1%. Patients with aortic prosthesis showed higher survival than patients with double implant (mitral and aortic) (p=0.026). Of the factors adjusted for mortality, only functional class and chronic renal failure showed statistically significant association. The incidence of PVT was 0.24/100 patients/year, and the first event occurred more than 1000 days after the implant. Smoking and pannus formation were significantly associated with PVT. No differences were found in INR variability between patients with and without thrombosis by prosthetic position, but significant differences were found in INR before thrombosis as compared with patients without thrombosis (INR= 2.20 [1.80-2.20] vs. 2.80 [2.20-3.40]; p= 0.040). The incidence of stroke and bleeding was 4.4% and 5.2% respectively.

**Conclusions:**

The study population was young, and rheumatic valve disease was common in this group. The prevalence of PVT was similar to that described in the literature, despite the low income and low educational level of our sample.

## Introduction

Valvular heart diseases (VHD) are highly prevalent, affecting more than 100 million people in the world.^1^ In developing countries, like Brazil, VHD account for a large proportion of hospitalizations for cardiovascular diseases. In these countries, rheumatic valve diseases are preponderant^2^ and represent a public health problem, with a greater impact on poorer and younger populations.^3^

Despite effective pharmacotherapy in most cases, more severe cases of VHD may be absolute indication for surgical therapy. Surgical procedures involve valve repair or replacement by implantation of a mechanical or a biological prosthetic valve. Due to their higher durability, mechanical prostheses have been widely preferred in younger patients.^4,5^ However, as compared with biological prosthesis, mechanical valve prostheses have been associated with a higher probability of thrombus formation and thromboembolic events due to their physical properties, with an overall incidence of mechanical valve thrombosis of 0.4 per 100 patients per year. It is of note that the incidence of thrombosis of mitral valve prosthesis is 0.5 per 100 patients per year, approximately five times more frequent than in the aortic position (0.1 per 100 patients per year).^6^

Thus, a careful postoperative follow-up of patients undergoing surgical valve replacement should be performed, since inadequate anticoagulation may lead to PVT and consequent dysfunction, with or without thromboembolism.^7^ Therefore, the choice for a mechanical prosthesis should be individualized even in younger patients, considering the variables: risk of bleeding, patient’s level of education and understanding, place of residence and distance from health facilities, patient’s preference and desire to become pregnant in women.^4^

There are few studies describing prosthetic thrombosis and its management in Brazil.^8,9^ Thus, the present study aimed to describe demographic, clinical, surgical characteristics and outcomes of patients undergoing mechanical heart valve replacement in a public tertiary hospital referral for cardiovascular care for users of the Brazilian Unified Health System, with emphasis on the incidence of mechanical prosthesis thrombosis.

## Methods

### Study design

This was a retrospective cohort study. Patients were identified in the database of the Department of VHD and in the Registry of the Department of Surgery of a tertiary hospital. The variables of interest were collected from electronic and conventional medical records. All patients that underwent surgery and were followed in the institution were monitored for INR (international normalized ratio) every 4-6 weeks at the anticoagulation outpatient center. Patients who had been regularly followed-up and were lost to follow-up for 12 consecutive months were checked for death at the website for out-of-court services of Rio de Janeiro state, since all patients with mechanical valve prosthesis had scheduled visits in the outpatient clinic every six months.

### Study population

All adult patients who underwent mechanical valve prosthesis replacement in Instituto Nacional de Cardiologia, Rio de Janeiro. Between January 2011 and December 2017 were studied.

### Study variables

The study variables were: sex, age, socioeconomic status, comorbidities, medications, presence of atrial fibrillation, region of residence (in the state of Rio de Janeiro), etiology and type of the original lesion, NYHA functional class in the last visit, echocardiogram functional and hemodynamic data after valve implantation and current ones, valve position and prosthesis brand, level of anticoagulation by measurement of prothrombin time and consecutive INR measurements in the last six months, or before the diagnosis of valve thrombosis or death. The incidence of valvular thrombosis, stroke and bleeding was calculated, and the type of intervention and presence or not of pannus associated with thrombosis was assessed.

### Operational definitions

Prosthetic valve thrombosis (PVT) was defined as any thrombus attached to or near a functioning valve, in the absence of infection, which occludes part of the blood flow or interferes with valvular function.

A new PVT was defined as an episode of PVT occurring more than three months after therapeutic intervention in which thrombus resolution was documented, confirmed by clinical assessment and complementary tests.

Severe bleeding was considered in case of an immediate risk of death, regardless of emergency surgical intervention or use of blood derivatives; major bleeding was defined as potentially severe bleeding, with mandatory hospitalization but predominantly conservative management, with or without the use of blood derivatives.

### Data analysis

Data were expressed as frequency (categorical variables), mean and standard deviation (continuous variables with normal distribution) or median and interquartile range (continuous variables without normal distribution). Statistical analysis was performed using the Jamovi software, version 1.2.2. Categorical variables were analyzed by the chi-square test and the Fisher’s exact test. The unpaired t-test was used for comparison of continuous variables with normal distribution, and the Mann-Whitney test for continuous variables without normal distribution. Comparison of means between more than two time points was made by analysis of variance (ANOVA) for repeated measures. Analysis of events (death and prosthetic thrombosis) was made using Kaplan-Meier curves. A p<0.05 was considered statistically significant. Adjusted hazard ratios (aHR) and their respective 95% confidence interval (CI) were estimated by Cox proportional hazard regression for analysis of the effects of variables on survival after valve replacement surgery. The assumptions of proportional hazards for the adjustments of the Cox regression model were tested by correlation analysis and the chi-square test based on scaled Schoenfeld residuals and transformed survival times.

### Ethical issues

The study was approved by the Instituto Nacional de Cardiologia ethics committee on August 01, 2018 (CAAE: 87442918.3.0000.5272, approval number 2.793.851).

## Results

In the study period, the total of 1,901 valve replacements were performed in the institution, including both biological and mechanical prostheses. A total of 473 (24.9%) patients received metallic prostheses (Figure 1), and 456 of these were followed-up until December 2019, with a mean follow-up of 4.4 years per patient. Seventeen patients lost follow-up in the institution.

**Figure 1:** Flowchart of inclusion of patients with valve prostheses and outcomes according to the location of the valve prosthesis.

A total of 609 mechanical prostheses were implanted, 49.9% of them in the aortic position, 30.2% in the mitral and aortic positions, and 19.9% in the mitral position. Protheses’ brands were St. Jude^R^ (Minneapolis, USA), (n=465, 74,2%), ATS Medical^R^ (Minnesota, USA) (n=159, 25,4%), Carbomedics^R^ (Austin, USA) (n=1, 0,2%). The models were not specified in 0.3% of the cases (n=2).

Table 1 presents clinical and demographic characteristics of the 473 study patients according to prosthetic position. Mean age was 46.9 ± 11.3 years. Most patients had (some or completed) elementary school, and family income until three minimum wages. Among the comorbidities, essential systemic arterial hypertension was the most common, detected in more than 50% of patients, followed by dyslipidemia in approximately one fourth, and type 2 diabetes mellitus. Of all patients, 46.5% were from Rio de Janeiro, and 37.3% were from Baixada Fluminense region.

**Table 1:** Demographic and clinical characteristics of patients undergoing mechanical valve replacement from January 2011 to December 2017

Variables			Aortic (n=236)	Mitral (n=96)	Mitral-aortic (n=141)	p value
(n = 473)	Male		148(62.7%)	31 (32.3%)	51 (36.2%)	p<0.01
	Female		88 (37.3%)	65 (67.7%)	90 (63.8%)	
**Age (years)** (n = 473)	< 20		0	0	0	p=0.015
	20-29		6 (2.5%)	2 (2.1%)	3 (2.1%)	
	30-39		26 (11.0%)	7 (7.3%)	14 (9.9%)	
	40-49		35 (14.8%)	19 (19.8%)	43 (30.5%)	
	50-59		70 (29.6%)	36 (37.5%)	46 (32.6%)	
	60-69		82 (34.7%)	25 (26.0%)	32 (22.7%)	
	≥ 70		17 (7.2%)	6 (6.2%)	3 (2.1%)	
**Educational attainment** (n = 382)	Illiterate		1 (0.42%)	0	1 (0.7%)	p=0.003
	Elementary school		95 (40.2%)	55 (57.3%)	77 (54.6%)	
	High school		73 (30.9%)	16 (16.6%)	32 (22.7%)	
	Higher education		22 (9.3%)	1 (1.0%)	9 (6.3%)	
**Family income** (minimum salaries) (n = 153)	< 1		23	1	15	p=0.697
	1 - 2		19	2	19	
	2 - 3		17	0	12	
	> 3		29	1	15	
Comorbidities	SAH	**Yes**	143(60.6%)	48 (50.5%)	66 (46.8%)	p=0.017
		**No**	93 (39.4%)	47 (49.5%)	75 (53.2%)	
	DLP	**Yes**	78 (33.6%)	25 (26.6%)	19 (13.7%)	p<0.001
		**No**	154(66.4%)	69 (73.4%)	120 (86.3%)	
	DM2	**Yes**	31 (13.6%)	16 (17.2%)	8 (5.8%)	p=0.020
		**No**	197(86.4%)	77 (82.8%)	129 (94.2%)	
	Smoking	**Yes**	19 (8.0%)	3(3.1%)	10 (7.1%)	p=0.273
		**No**	217(92%)	92 (96.9%)	131 (92.9%)	
	Previous stroke	**Yes**	5 (2.1%)	9(9.5%)	12 (8.5%)	p=0.006
		**No**	229(97.9%)	86 (90.5%)	129 (91.5%)	
	TIA	**Yes**	2 (0.8%)	0	1 (0.7%)	p=0.672
		**No**	232(99.2%)	95 (100%)	139 (99.3%)	
	CRF	**Yes**	4 (1.7%)	7 (7.3%)	5 (3.6%)	p=0.038
		**No**	232(98.3%)	89 (92.7%)	135 (96.4%)	
	COPD	**Yes**	12 (5.1%)	2 (2.1%)	3 (2.1%)	p=0.224
		**No**	223(94.9%)	93 (97.9%)	137 (97.9%)	
	Alcohol consumption	**Yes**	1 (0.4%)	0	0	p=0.605
		**No**	233(99.6%)	95 (100%)	140 (100%)	
	Hepatopathies	**Yes**	0	0	3 (2.1%)	p=0.029
		**No**	233 (100%)	94 (100%)	137(97.9%)	
**Atrial fibrillation** (n = 473)	Present		19 (8.0%)	52 (54.2%)	62 (44%)	p<0.001
	Absent		217 (92%)	44 (45.8%)	79 (56%)	

SAH: systemic arterial hypertension; DLP: dyslipidemia; DM2: type 2 Diabetes Mellitus; TIA: transient ischemic attack; CRF: chronic renal failure; COPD: chronic obstructive pulmonary disease. Note: Numbers in educational attainment, family income, and comorbidities correspond to those with data available in the medical charts, as this was a retrospective study.

Rheumatic valvular disease was the most common etiology (57.7%), followed by degenerative valve disease (12.9%) and bicuspid aortic valve (12.1%). Infective endocarditis was the main secondary etiology, leading to a second valve replacement surgery in 24 (5.1%) of the cases. Figure 2 shows the etiologies by categories (mitral, aortic and double valve replacement, *i.e.*, mitral and aortic).

**Figure 2:** Etiology of heart valve disease by position of the mechanical valve prosthesis implanted.

The following severe lesions were found: 107 aortic stenosis (45.9%), 93 (39.9%) aortic insufficiency and 16 (6.8%) double aortic arch; severe mitral stenosis in 41 (44%), mitral insufficiency in 30 (32.2%) and double valve in 8 (8.6%).

The New York Heart Association (NYHA) functional class (FC) of 422 patients reported in the last visit at the outpatient was FC I in 323 (76,5%); FC II in 85 (20,1%), FC III in 12 (2,8%), and FC IV in only two (0.5%).

Table 2 describes echocardiographic data post-valve replacement and recent data obtained during patients’ follow-up. As compared with the echocardiogram performed right after surgical implantation, more recent tests revealed that aortic prostheses showed improvement of the hemodynamic parameters (p<0.001). In mitral position and double replacement (mitral and aortic positions), there was an improvement in the mean ejection fraction and mean pressure gradient between the left atrium and left ventricle.

**Table 2:** Comparison of echocardiographic parameters between after implant and recent parameters by mechanical prothesis position

		PRIOR TO HOSPITAL DISCHARGE (mean [SD] or median [IQR])	RECENT RESULTS (mean (SD) or median [IQR])	p-values
**Aortic valve prosthesis** (n= 233)				
	LVEF (%)	54.1 (14.7)	62.6 (12.0)	< 0.001
	Maximum gradient LV/AO (mmHg)	32.0 [25.0 - 41.8]	26.0 [20.0 - 34.0]	< 0.001
	Mean gradient LV/AO (mmHg)	18.0 [13.0 - 23.0]	14.0 [11.5 - 15.8]	< 0.001
**Mitral valve prosthesis** (n= 93)				
	LVEF (%)	54.2 (2.7)	56.8 (13.4)	0.028
	Maximum gradient LV/AO (mmHg)	13.3 (4.55)	26.6 (4.04)	0.837
	Mean gradient LV/AO (mmHg)	5.0 [4.0 - 6.0]	4.0 [4.0 - 5.0]	0.036
**Mitral and aortic prosthesis** (n= 141)				
	LVEF (%)	55.5 (14.2)	61.2 (12.7)	<0.001
	Maximum gradient LV/AO (mmHg)	30.0 [23.0 - 39.5]	29.0 [21.0 -40.5]	0.477
	Mean gradient LV/AO (mmHg)	17.0 [11.0 -23.0]	16.0 [11.0 -22.3]	0.642
	Maximum gradient LV/AO (mmHg)	12.4 (5.05)	12.4 (5.1)	0.749
	Mean gradient LV/AO (mmHg)	5.0 [3.0 - 6.0]	4.0 [3.0 - 5.0]	0.003

LVEF: left ventricular ejection fraction; gradient LV/AO: pressure gradient between the left ventricle (LV) and the aorta; unpaired t-test and Mann-Whitney test.

Figure 3 shows the survival curve for the implanted prosthesis by position. Patients with aortic prosthesis showed higher survival than patients with prosthesis in the mitral and aortic position (p=0.026). No other differences were seen in the other comparisons. In the survival curve, no statistical difference was seen between age groups or sex (Supplementary Figures 1 and 2). When we analyzed survival rates in patients with rheumatic valvular disease only, no difference was observed between valve positions. However, in non-rheumatic patients, a difference was observed between valve positions, although the number of individuals undergoing mitral valve surgery or both mitral and aortic valve surgery was very small. Most non-rheumatic patients had undergone aortic valve replacement (n=127) (Supplementary Figures 3 and 4). No difference was found in survival between rheumatic and non-rheumatic patients with aortic valve prosthesis.

**Figure 3:** Survival curve according to valve position in patients who had surgery with mechanical valve prosthesis, Instituto Nacional de Cardiologia, Rio de Janeiro, January 2011 to December 2017.

Cox regression for the variables associated with survival revealed that the main factor related to death was functional class after valve implantation. For FC II, aHR was 5.18 (2.17-12.39; p<0.001), for FC III, aHR was 41.13 (14.95-113.15; p<0.001) and for FC IV, aHR was 200.48 (21.60-1861.12; p<0.001). Another associated factor was the presence of chronic renal failure, with an aHR of 3.52 (1.12-11.09, p=0.032). The positions of mechanical valve prosthesis did not show statistically significant difference after HR adjustment. These results are presented in Supplementary Figure 6.

Seventy-six (16.1%) all-cause deaths occurred, 36 (7.4%) within 30 days. Considering the number of deaths by prosthesis position, mortality rate among patients with aortic prosthesis was 6%, mitral prosthesis 8.2%, and mitral and aortic position 14.2%. The most frequent causes of death were cardiogenic shock (R57.0), hypovolemic shock and disseminated intravascular coagulation (R57.1 and D65). Of the 17 patients who were lost to follow-up, one patient died according to the website for out-of-court services of Rio de Janeiro state, but the cause of death was not specified.

The overall incidence of thrombosis per patient was 1.1% (0.24 per 100 patients per year), with seven thrombotic events in five patients. Mechanical PVT occurred in the late post-implantation period, with the first being registered only five years after surgery. Details of the patients who had PVT are described in Chart 1.

**Chart 1:** Clinical and echocardiographic characteristics, and outcomes of five patients (and seven events) diagnosed with mechanical prosthetic valve thrombosis

Patient	1	2	3	4	5
Age	43	44	52	38	54
Sex	Male	Female	Male	Female	Female
Educational level	Elementary school	Elementary school	Elementary school	Superior	Elementary school
Family income	2 MSs	1 MS	5MSs	5MSs	4MSs
Rio de Janeiro	Yes	Yes	Yes	No	Yes
Primary etiology	Rheumatic	Rheumatic	Rheumatic	Rheumatic	Congenital - biscuspid
Prothesis	Mitral 27	Aortic 18 Mitral 27	Aortic 21 Mitral 27	Aortic 21 Mitral 29	Aortic 19
Prothesis brand	ATS	ATS	St Jude ATS	St Jude St Jude	St Jude
Atrial fibrillation	Yes	No	Yes	No	No
Time of implant x thrombotic event	8 years	A – 5 years B – 7 years	A – 5 years B – 2 years	6 years	5 years
Smoking	No	No	Yes	Yes	No
NYHA functional class	III	A – I B – III	A – II B – IV	III	II
Follow-up	Current	Current	Current	Current	Not current (death)
LV dysfunction	Yes	Yes	No	No	No
Pannus	No	Yes	Yes	Yes	Yes
Treatment	UH + TT	A – UH + TT B – Surgery	A – UH + TT B – Surgery	Surgery	Surgery

A and B refer to the first and second episodes of prosthetic valve thrombosis, respectively, in the same patient; NYHA: New York Heart Association; ATS: Medtronic’s bileaflet mechanical prosthesis; St. Jude: Abbott’s bileaflet mechanical prosthesis; UH: unfractionated heparin; TT: thrombolytic therapy; LV (left ventricular) dysfunction: degree of left ventricular failure characterized by ejection fraction ≤ 52% (Teichholz method), according to the American Society of Echocardiography; MS: minimum salary.

Table 3 shows the comparison of monthly variation in INR, from the most recent (INR6) to the earliest (INR1), between patients with and without PVT. There was no significant variation between these groups over time. The absence of values between parentheses indicates the absence of standard deviation values, since there was only one patient with thrombosis in the sample. Similarly, no difference was found in variability of INR between patients with bleeding as compared with those without bleeding.

**Table 3:** Variability of international normalized ratio (INR) between patients with and without prosthetic valve thrombosis

Thrombosis	INR month 1 (n=52)	INR month 2 (n=144)	INR month 3 (n=256)	INR month 4 (n=335)	INR month 5 (n=381)	INR month 6 (n=407)	p-value
**Yes** (n=5)	4.70 (-)	1.50 (-)	2.37 (0.61)	3.00 (2.02)	2.30 (0.673)	2.06 (0.42)	0.392
**No** (n=451)	3.09 (1.45)	3.15 (1.30)	2.97 (1.12)	2.97 (1.23)	2.82 (1.00)	2.94 (1.26)	

Values expressed as mean (±SD); INR: international normalized ratio; ANOVA with repeated measures.

Table 4 describes INR values obtained from six collections before the thrombotic event of the patients who had PVT, and from six collections before the last visit in those who did not have PVT. No significant difference was found in INR values between patients with and without thrombosis regarding the position of the prosthetic valve. Values of INR of some months of patients with mitral and aortic position were missing, which made comparisons difficult. INR of patients who had thrombosis was significantly lower than of patients who did not have thrombosis (median 2.20 [1.80-2.20] and 2.80 [2.20-3.40], respectively, p=0.04) in the month prior to the event (INR6), independently of the valve prosthesis position. In the analysis of each prosthesis position, no statistically significant difference was found in INR between patients with PVT and those without PVT (Table 4). We found an association between smoking habit and PVT (2 of 5 patients with PVT vs. 27 of 441 patients without PVT). The presence of pannus was detected at surgery in 11 patients, four of them associated with thrombosis (p<0.001). Of seven events of PVT detected in five patients, one death occurred in the immediate postoperative period of valve replacement surgery (Chart 1).

**Table 4:** Comparative analysis of monthly INR between patients with and without thrombosis by position of the mechanical valve prosthesis

Prosthesis /Position		Mitral (target INR=2.5 – 3.5)*		Mitral and aortic (target INR=3)*		Aortic (target 2.5 – 3.5)*	
Thrombosis		Yes	No	Yes	No	Yes	No
**INR month 1**		---------	---------	4.70 [4.70 - 4.70]	2.80 [2.55 - 4.00]	---------	---------
	p value	---------	---------	0.193		---------	---------
**INR month 2**		---------	---------	1.50 [1.50 - 1.50]	3.15 [2.32 -3.60]	---------	---------
	p value	---------	---------	0.163		---------	---------
**INR month 3**		---------	---------	2.70 [2.60 - 2.80]	3.00 [2.30 - 3.50]	1.70 [1.70-1.70]	2.70 [2.10-3.30]
	p value	---------	---------	0.600		0.161	
**INR month 4**		---------	---------	1.85 [1.68 - 2.03]	2.95 [2.40 - 3.73]	5.30 [5.30-5.30]	2.60 [2.20-3.20]
	p value	---------	---------	0.063		0.098	
**INR month 5**		1.30 (-)	2.94 (0.981)	2.60 (0.141)	3.05 (1.10)	2.70 [2.70-2.70]	2.60 [2.00-3.10]
	p value	0.100		0.565		0.865	
**INR month 6**		2.60 [2.60-2.60]	3.00 [2.2-3.70]	2.20 [1.85 - 2.2]	2.90 [2.20 - 3.50]	1.80 [1.80-1.80]	2.60 [2.10-3.30]
	p value	0.71		0.073		0.211	

INR reference range as proposed by Nishimura et al.^27^; unpaired t-test and Mann-Whitney test. INR: international normalized ratio.

Regarding hemorrhagic events, 23 patients with bleeding were found, eight (1.7%) with severe bleeding and 15 (3.5%) with major bleeding. Bleeding rate was 1.02 per 100 patients per year. There were two deaths among patients with severe bleeding, one caused by cardiac tamponade and caused by hemorrhagic stroke. Ischemic stroke occurred in 4.4% of the sample, with an incidence of 0.86 per 100 patients per year.

Five patients with periprosthetic leak were identified. Two of them had had infective endocarditis as the cause of mechanical valve replacement, two patients developed periprosthetic leak after valve replacement, and one had rheumatic mitral and aortic valve disease. Only one patient required surgery and died; percutaneous occlusion was performed in two patients.

## Discussion

The present study evaluated demographic, clinical, surgical characteristics, and the outcomes of patients undergoing implantation of mechanical valve prosthesis in a public tertiary hospital referral for cardiovascular care for users of the Brazilian Unified Health System, with emphasis on the incidence of mechanical prosthesis thrombosis. In this sample of 473 patients, we found a similar proportion of men and women, which is in accordance with recent literature,^10,11^ but different from the study by Brandão et al.^9^ conducted in the state of São Paulo and published three decades ago, in which male sex corresponded to 64.3%. Most patients had a low socioeconomic status, with a monthly income up to three minimum wages, and some or completed elementary school. Mean age of our patients was 47 years, lower than that described in the international literature,^12–14^ but similar to Brazilian studies,^10,11^ explained by the fact that rheumatic disease was the main cause of heart valve replacement in the Brazilian public health system. Most patients were from the city of Rio de Janeiro, followed by the metropolitan area I (Baixada Fluminense). The importance of the place of residence is related to the possibility of better access to a good follow-up in the outpatient anticoagulation clinic.

Rheumatic valve disease was the most common primary etiology, affecting more than half of patients. This is in accordance with national data, such as the study carried out in the city of Salvador, and a article on data of valve replacement surgery among users of the public health system, which is in contrast with studies in developed countries.^1,2,7^ Data from the Brazilian literature, of populations with characteristics similar to our sample, show a predominance of mitral valve surgery, which is different from our study that revealed a predominance of aortic valve surgery. We believe that many factors are responsible for this difference, including i) rheumatic valvular disease frequently results in mitral stenosis, specially in female patients; in this group, there is a preference for bioprosthesis implantation in reproductive-age women; ii) although percutaneous balloon mitral valvuloplasty or commissurotomy (without valve replacement) is a possible therapeutic procedure in case of mitral valve disease, the approach is not feasible in rheumatic aortic valve disease. In fact, in our sample, there were cases of mechanical mitral valve replacement prior to the study, but during the study, only aortic valve replacement was performed. Of the 67 rheumatic patients that underwent aortic valve replacement, mitral commissurotomy associated with mechanical aortic valve replacement was performed in seven (10.4%) patients. There were mild rheumatic mitral lesions associated with the aortic valve replacement in 34 patients (50.7%), and two patients had concomitant moderate mitral valve disease, who did not undergo intervention at surgery. The ATS and St. Jude prostheses were the almost exclusive brands used. Patients with aortic valve implantation showed higher survival than patients undergoing double (aortic and mitral) valve replacement (p=0.026), which corroborates previous studies.^13,15^ No differences were found in survival between sex and age groups, although there were only 10 patients (17.1%) older than 65 years in our sample. Our results were different from a Brazilian study^10^ that reported a higher survival among female than male patients in the first and fifth year of follow-up.^10^ According to the multivariate analysis, the factors strongly correlated with death after valve replacement was cardiac function in the outpatient follow-up and presence of chronic renal failure as comorbidity.

In our study, overall mortality and 30-day mortality were 16% and 7.4%, respectively, with the highest rates among patients with double valve replacement of the aortic and mitral valves. In the Japanese study by Tominaga et al.,^16^ published in 2005, in a 10-year follow-up of patients who underwent valve replacement with the Carbomedics bileaflet mechanical prosthesis, the authors reported an early mortality rate for the total population of 2.8% (1.2% for the aortic position, 3.6% for the mitral position, and 3.8% for double valve implants). A Swiss article published in the ‘90s^13^ and a Belgian study^14^ described an in-hospital mortality of 5.7% and 5.2%, respectively, which were lower than ours. It is worth mentioning that the populations of these studies were very different, with a predominance of older patients. A Brazilian study^11^ of hospitals of the public health system reported a mortality rate of 22.1%, higher than that found in our study. In a two-year period, the authors found a mortality rate of 12.3%, 8.5% of them among patients with aortic valve prosthesis, 12.2% among patients with mitral valve prosthesis, and 18.4% in patients with double valve implants. In our study, the five-year follow-up survival rate was 83.4% versus 74.5% in this Brazilian study.^11^

Considering the occurrence of PVT, our data are in accordance with the international literature, of 0.1 – 5.7% and 0.3-1.3%, respectively.^7,17^ Put in a different way, the incidence of thrombosis in our in the study was slightly lower than that reported by Van Nooten et al. (0.31 per 100 patients per year), indicating the low incidence of this event in our institution. The mean time to the first thrombosis event in our study was longer than that in the Canadian study (39 months).^18^ Considering the low socioeconomic status of our population, the low incidence of thrombosis is a positive result, which encourages us to consider the recommendation of mechanical prosthesis for younger patients with less fear.

Smoking habit, which was shown to be significantly associated with PVT in our study, was identified from patients’ medical charts, and is known to be a risk factor for a secondary hypercoagulable state, as it contributes to a pro-thrombotic mechanism, as described in the literature.^7^

The diagnosis of mechanical PVT was made based on clinical assumptions and use of complementary tests available at the institution. Among these tests, in our opinion, transesophageal echocardiography is the most important tests. Radioscopy has been shown to be useful as a complementary diagnostic method to echocardiography. Anticoagulation strategies after mechanical valve replacement have not been well established, with differences between European and American guidelines. The American guidelines report complications related to fluctuations in INR with warfarin, suggesting the adoption of a unique INR target.^19^ For aortic valve prosthesis, a target INR of 2.5 was suggested; for mitral or aortic prosthesis and presence of risk factors (atrial fibrillation, previous thromboembolism, left ventricular dysfunction, hypercoagulable state), target INR of 3.0 combined with aspirin at a dose of 75-100 mg (class IA). The European guidelines determine INR values according to thrombogenicity of the prosthesis and presence of risk factors, and combination of aspirin in case of concomitant atherosclerotic disease and/or thromboembolism despite inadequate INR.^20^ Considering the target INR in the guidelines, not only considering the antithrombotic effect but also the bleeding factor, only 40.6% of our patients with mitral and/or aortic valve prosthesis had INR within the target range, based on laboratory results in the six months prior to the last medical visit. There are several articles discussing the challenge of maintaining the anticoagulation target ranges.^21–23^ Variability in INR values in the last six months were not statistically different between patients with and without PVT, or between prosthesis position. Nevertheless, when all patients with PVT were compared with those without PVT, we observed a significant difference in the last INR. Although expected, further differences between these groups may have not been possible due to the small number of events in the sample.

Pannus formation was significantly associated with the presence of thrombus. This finding corroborated the literature, as many studies have suggested that thrombosis occurs concomitantly with other causes of valve prosthesis dysfunction, such as pannus growth. The presence of pannus is a pro-thrombotic factor.^7^

With respect to treatment and outcomes of patients with PVT, three of the five patients received pharmacotherapeutic treatment with unfractionated heparin followed by thrombolytic therapy. There are articles reporting the effectiveness of long-term oral anticoagulation therapy combined with unfractionated heparin in preventing early thromboembolic events in obstructive and small (<5mm) thrombi after mitral valve replacement.^24^ There is a consensus for this therapy for non-obstructive small thrombi in the left side of the heart, still with reduced effectiveness and recurrence of thrombosis in 16%.^25^ Heparin seems ineffective for treatment of obstructive thrombus.^26^ In our study, three patients underwent thrombolytic therapy with recombinant tissue plasminogen activator (rT-PA). All patients showed a good response, with recurrence of thrombosis in two patients in a time interval of seven months and two years, respectively. Our small sample precludes a more accurate comparison with literature data. In addition, unfractionated heparin was ineffective in our patients. American and European guidelines state that surgical treatment is the first option for patients with mechanical PVT and NYHA III /IV functional class except for those at high surgical risk (class IIa). Data from the literature have supported the use of thrombolytic therapy for PVT, including the TROIA and PROMETEE,^27^ using, respectively, slow-infusion and ultra-slow infusion of rT-PA. The surgical approach for thrombosis of mechanical valve prosthesis is a procedure of valve replacement and its risks cannot be underestimated; there are reports of mean mortality rates of 12% in these circumstences.^28^ Surgery in emergency or urgent care has been the strategy of choice, although associated with high mortality (7.1-69%), depending on patients’ functional status.^29^ For two patients in our study surgery was the first option, and in one patient, pregnancy and infective endocarditis were detected.

The rate of bleeding in our study was similar to that reported in an Italian study published in 2018.^20^ All patients required hospitalization and specific treatment and follow-up. In a Brazilian study, patients undergoing mechanical valve prosthesis replacement were followed-up for 40.6 months, and the incidence of bleeding was 0.95%/patient-year.^9^ Another study reported that in patients with INR of > 2.5 to 4.5 taking warfarin, the likelihood of bleeding is 3%/patient-year.^29^

Regarding ischemic stroke, the incidence was similar to that in the literature, 0.9-3.6 per 100 patients per year.^21^

Limitations of the study include the fact that this was a single-center study, conducted with public health users, so that the conclusions may not be applicable to other centers. Due to the retrospective nature of the study, some data could not be obtained. Besides, the number of events observed was small, including PVT and bleeding, which limit the analysis of variables possibly associated with these events. Another possible limitation is missing events (PVT or bleeding) that may have occurred in other hospitals.

## Conclusions

Our patient population was young, with history of surgical valve replacement, and rheumatic heart disease as its main cause.

The incidence of PVT was 1.1%, which is in accordance with the international literature, with late events after the implant. Due to the low educational level and income of our patients, this was a positive result, which encourages us to recommend implantation of mechanical valve prosthesis for these patients.

Factors associated with PVT in all valve positions assessed together were INRs are out of the target range, smoking and presence of pannus. The factors strongly correlated with death after valve replacement was cardiac function in the outpatient follow-up and presence of chronic renal failure as comorbidity.

## *Supplemental Materials

For additional information Supplemental Material 1, please click here.

For additional information Supplemental Material 2, please click here.

For additional information Supplemental Material 3, please click here.

For additional information Supplemental Material 4, please click here.

For additional information Supplemental Material 5, please click here.

For additional information Supplemental Material 6, please click here.
